# Infectious Complications of Targeted Therapies for Solid Cancers or Leukemias/Lymphomas

**DOI:** 10.3390/cancers15071989

**Published:** 2023-03-27

**Authors:** Benoît Pilmis, Yousra Kherabi, Pauline Huriez, Jean-Ralph Zahar, Djamel Mokart

**Affiliations:** 1 Equipe Mobile de Microbiologie Clinique, Groupe Hospitalier Paris Saint-Joseph, 75014 Paris, France; 2 UMR 1319, Institut Micalis, Université Paris-Saclay, INRAeChâtenay Malabry, AgroParisTech, 92290 Chatenay Malabry, France; 3 Infection Control Unit, AP-HP Hôpital Avicenne, Université Sorbonne Paris Nord, 93000 Bobigny, France; 4 Medical Surgical Intensive Care Unit, Institut Paoli Calmettes, 13009 Marseille, France

**Keywords:** immune checkpoint inhibitors, Bruton’s tyrosine kinase inhibitor, CAR-T cell therapy

## Abstract

**Simple Summary:**

Targeted therapies have revolutionized the management of hematological malignancies and solid organ cancers. These new treatments present numerous infectious complications. We aim to present the main infectious complications related to immune checkpoint inhibitors, Bruton’s tyrosine kinase (BTK) inhibitors, phosphatidylinositol 3-kinase inhibitors (PI3K), antiapoptotic protein BCL-2 inhibitors, Janus kinase inhibitors and CAR-T cell infusion treatments. The knowledge of complications allows the physician to better identify patients at risk in order to implement diagnostic and therapeutic strategies, or to discuss the implementation of preventive measures.

**Abstract:**

Background: Infections are well known complications of some targeted drugs used to treat solid organ cancer and hematological malignancies. Furthermore, Individual patient risk factors are associated with underlying pathologies, concomitant immunosuppressive treatment, prior treatment and use of anti-infective prophylaxis. Immune-related adverse events (irAEs) are frequent among patients treated with new targeted drugs. Objectives: In this narrative review, we present the current state of knowledge concerning the infectious complications occurring in patients treated with immune checkpoint inhibitors (ICIs), Bruton’s tyrosine kinase (BTK) inhibitors, phosphatidylinositol 3-kinase (PI3K) inhibitors, antiapoptotic protein BCL-2 inhibitors, Janus kinase inhibitors or CAR-T cell infusion. Sources: We searched for studies treating infectious complications of ICIs, BTK inhibitors, PI3K inhibitors, antiapoptotic protein BCL-2 inhibitors and CAR-T cell therapy. We included randomized, observational studies and case reports. Content: Immune-related adverse events (irAEs) are frequent among patients treated with new targeted drugs. Treatment of irAEs with corticosteroids and other immunosuppressive agents can lead to opportunistic infections. Bruton’s tyrosine kinase (BTK) inhibitors are associated with higher rate of infections, including invasive fungal infections. Implications: Infections, particularly fungal ones, are common in patients treated with BTK inhibitors even though most of the complications occurring among patients treated by ICIs or CART-cells infusion are associated with the treatment of side effects related to the use of these new treatments. The diagnosis of these infectious complications can be difficult and may require extensive investigations.

## 1. Introduction

Over the past two decades, there has been a tremendous shift in cancer treatment from broad-spectrum cytotoxic drugs to targeted drugs. As early as 1909, Paul Ehrlich predicted that the immune system normally prevents the formation of carcinomas. Malignant neoplasms are known to downregulate major histocompatibility complex (MHC)-I molecules, preventing recognition of tumor cells by cytotoxic T lymphocytes (CTLs). Recent advances in treatment aim to provide effective immunotherapy with minimal toxicity. Therefore, cancer immunotherapy aims to harness the memory and specificity of the immune system to effectively eliminate malignant neoplasms. Current therapeutic approaches include cytokine therapy, CAR-T cell therapy and checkpoint inhibitor therapy. These new therapies are often associated with inflammatory and/or infectious complications requiring intensive care unit (ICU) admission. Among these new drugs, immune checkpoint inhibitors (ICIs), Bruton’s tyrosine kinase (BTK) inhibitors, phosphatidylinositol 3-kinase (PI3K) inhibitors, anti-apoptotic protein BCL-2 inhibitors, Janus kinase (JAK) inhibitors, or CAR-T cell infusion have been identified as frequently associated with life-threatening side effects or infectious complications (Table 1) [1].

These adverse inflammatory effects may require the introduction of immunosuppressive drugs such as corticosteroids, thereby increasing the risk of infection. In addition, the differential diagnosis of inflammation/infection is often difficult.

In this article, we present a narrative review, from an infectious disease perspective, of the safety profile of oral and parenteral drugs used to treat solid organ and hematologic malignancies. We analyze the infectious complications associated with these innovative therapies, including ICIs, CAR T cells, BTK inhibitors, JAK inhibitors, and PI3K inhibitors (Table 2).

## 2. Material and Methods

A PubMed search was conducted to identify studies of agents currently used to treat solid organ and hematologic malignancies that reported infectious events. The search focused on systematic reviews, meta-analyses, clinical trials, guidelines, and case reports, with an emphasis on those agents considered most relevant to clinicians and a selection of drugs with a greater impact on the risk of infection. The selection of molecules was based on those most frequently associated with serious infectious adverse events leading to hospitalization in intensive care units, as well as our clinical experience and expertise.

## 3. Monoclonals Antibodies

Work on the activity of monoclonal antibodies on tumor cells began in the 1970s. Destruction of target cells by monoclonal antibodies (mAbs) can be achieved in several ways, including immune-mediated cell killing, direct antibody action (blocking of receptors or delivery of the target toxic agents), tumor environment and specific antibody action on the vascular system.

### Immune Checkpoint Inhibitors

Checkpoint inhibitor immunotherapies (also known as immune checkpoint inhibitors (ICIs)) are immunomodulatory antibodies used to boost the immune system. Over the past decade, the development of immune checkpoint blockade antibodies, such as those directed against cytotoxic T-lymphocyte antigen 4 (CTLA-4), programmed death receptor 1 (PD-1) and programmed death ligand 1 (PD-L1), has shown great results in the treatment of melanoma and other cancers making it a reference treatment for melanoma and other cancers. The anti-CTLA-4 antibody ipilimumab was the first immune checkpoint antibody approved for the treatment of patients with advanced melanoma [2,26,27].

The first marketed human IgG4 anti-PD-1 checkpoint inhibitor antibodies were pem-brolizumab and nivolumab. Their main indication was the treatment of refractory and unresectable melanoma [28,29,30]. ICIs are now indicated as first-line treatment for these pathologies [31,32,33,34]. Phase 2 randomized controlled trials evaluating the safety of CTLA-4-targeted agents [2,3,4] or (PD)-1 and (PD-L1)-targeted agents in patients with advanced melanoma did not suggest an increased risk of infection.

However, the use of immune checkpoint blockade drugs is associated with the occurrence of numerous adverse events related to the stimulation of the immune system. These side effects affect many organs (lungs, pancreas, liver, etc.). Treatment of these side effects is based on symptomatic treatments for benign forms and low or high dose corticosteroids or even the use of tumor necrosis factor alpha (TNF-α) inhibitors (infliximab), azathioprine and mycophenolate mofetil. Among patients taking anti-CTL4 agents, 70% develop adverse events, 20% of which are severe [35]. In addition, 30% of patients treated with anti-CTLA-4 develop an infectious complication [36]. In patients treated with anti-PD-1 or anti-PD-L1, 80% develop adverse events, 8% of which are severe [35]. The safety profile of PD-1/PD-L1 targeted agents appears to be better than that of CTLA-4 targeted molecules, with a lower proportion of exposed patients developing severe irAEs. Indeed, irAEs appear to be less common in patients exposed to PD-1/PD-L1 targeted agents. However, the common combination of ipilimumab and a PD-1 inhibitor carries a higher risk of irAEs than either agent alone [26,37].

Adverse events occur primarily during the first 4 months of treatment with a median of 6 weeks for anti-CLTA-4 and 2.5 months for anti-PD-1/anti-PD-L1. Seventy percent of adverse events resolve with discontinuation of ICIs with a median of 4–8 weeks after discontinuation [38].

In a study of 740 patients treated with ICIs (73% anti-CTLA-4), 54 (7.3%) presented with a serious infectious episode (*n* = 58), mostly lower respiratory tract infections [39]. Of these, 46% received concomitant corticosteroid therapy and 16% received anti-TNF-alpha therapy. The most common infectious agents isolated in the included patients were bacterial (79.3%), fungal (10.3%), viral (8.6%) and parasitic (1.7%). Two cases of invasive pulmonary aspergillosis, three cases of *Pneumocystis jirovecii* pneumonia, one case of candidemia and one case of strongyloidiasis were reported. Serious infectious events were significantly more frequent in patients treated with corticosteroids (85% vs. 43%, *p* < 0.0001; OR = 7.71 (3.71–16.18)) or infliximab (24% vs. 6%, *p* < 0.0001; OR = 4.74 (2.27–9.45)).

In addition, there are several case reports that have also highlighted opportunistic infections with a variety of pathogens, including *Aspergillus fumigatus* [40,41,42,43,44,45], *Pneumocystis jirovecii* [41,46,47,48], John Cunningham (JC) virus [49,50,51], CMV [41,52,53] and *Campylobacter* [54]. These reports highlight the need to have a threshold for investigation of opportunistic infections after treatment of immune-related adverse events and consideration of *Pneumocystis jirovecii* prophylaxis. Indeed, *Pneumocystis jirovecii* prophylaxis should be considered when secondary immunosuppression is given for at least four weeks [55,56].

Tuberculosis reactivation was one of the first infections associated with immune checkpoint inhibitors to be described. Indeed, the PD-1/PD-L1 pathway plays a substantial role in tuberculosis physiopathology. PD-1/PD-L1 deficiency has been associated with an increase in TNF-α, IL-1 and IFN-γ and dysregulation of the innate immune system [27,28,29,30]. Thus, two mechanisms explain the risk of tuberculosis in patients treated with ICIs: (1) downregulation of the PD-1/PD-L1 pathway induces an exacerbated inflammatory response; (2) treatment of irAEs with corticosteroids and TNF- inhibitors favors the development of active tuberculosis [31,32]. In a recent study, patients treated with PD-1/PD-L1 inhibitors had an increased risk of active tuberculosis (OR = 1.79 (95% CI 1.42–2.26; *p* < 0.0001)). In addition, a Japanese study of 297 lung cancer patients treated with PD-1/PD-L1 inhibitors showed a 1.7% incidence of *Mycobacterium tuberculosis* reactivation. The infection developed between 22 and 398 days after the start of immune checkpoint inhibitor therapy. A recently published study prospectively evaluated the value of the interferon-gamma release assay (IGRA) in patients treated with ICIs for lung cancer. The test was performed prior to ICI and at 6 and 12 months. Of the 178 patients enrolled, 3 had IGRA reversal during immunotherapy and 4 had IGRA reversal. One of the four patients with IGRA conversion developed active tuberculosis. Physicians should be aware of the potential development of tuberculosis during ICI therapy, and IGRA testing is a useful tool to assess the risk of developing active tuberculosis [57].

ICI use may be associated with a risk of worsening chronic viral infections. A meta-analysis of 186 ICI-treated patients with chronic viral infections (HBV (*n* = 89) or HCV (*n* = 98)) found an increased risk of hepatic cytolysis in chronic liver infections, but no deaths from fulminant hepatitis.

## 4. Bruton’s Tyrosine Kinase Inhibitors

The BTK gene was discovered in 1993 and over 800 mutations in the BTK gene have been described. Most result in a deficiency in the production of the BTK protein. Ibrutinib, acalabrutinib and zanubrutinib are oral drugs that irreversibly inhibit Bruton’s tyrosine kinase (BTK) in the pathway or at the B-cell receptor (BCR). Stimulation of the transmembrane BCR protein leads to activation of several tyrosine kinases, including BTK and phosphatidylinositol 3-kinase (PI3K), which in turn activate proliferation and survival signals of B lymphocytes. Occupation of the BTK activation site by ibrutinib does not appear to have a direct effect on the normal B cell. B cells in chronic lymphocytic leukemia (CLL) or mantle cell lymphoma (MCL) differ from normal B cells in that they often have higher levels of ongoing BCR or other signaling pathway activity. This suggests that the effect of ibrutinib is likely to be minimal in normal B cells but marked in CLL or MCL cells.

BTK inhibitors are currently approved for the treatment of several lymphoproliferative disorders, including mantle cell lymphoma, chronic lymphocytic leukemia, Walden-Strom macroglobulinemia and marginal zone lymphoma [25,58].

One of the most common adverse effects observed in patients treated with Bruton’s tyrosine kinase inhibitors is infection. In a systematic review of ibrutinib clinical trials that included 48 studies and more than 2000 patients, infections (of any grade) were reported in 56% of patients treated with ibrutinib [59]. A more recent study found a cumulative incidence of 0.55 infections per 1000 person-days during the first year of targeted therapy, with higher rates in the first 3–6 months [60]. In a retrospective study of 378 patients receiving ibrutinib for chronic lymphocytic leukemia (CLL) or non-Hodgkin’s lymphoma, 43 (11.4%) patients developed serious infections. Of those with serious infections, 23 (53.5%) developed serious bacterial infections, 16 (37.2%) developed invasive fungal infections, and 4 (9.3%) developed viral infections [61]. The main infections reported in the literature are bacterial infections, especially those related to *Staphylococcus aureus.* Invasive fungal infections, although rarely reported in clinical trials, have also been associated with the use of ibrutinib in several observational studies [61,62,63]. The most common causative agents were *Aspergillus* spp. although non-Aspergillus infections including disseminated cryptococcosis, endemic fungal infections and *Pneumocystis jirovecii* pneumonia have also been reported [64,65,66]. Other rare infections such as tuberculosis and progressive multifocal leukoencephalopathy (PML) have also been reported [67].

According to the literature, one of the peculiarities of these infections is the non-neutropenic status of the patient at the time of diagnosis and the very early onset, typically during the first six months after initiation of treatment [61,68]. One possible explanation is inhibition of the BTK pathway in macrophages, which is involved in fungal defense [62,69]. The incidence of invasive aspergillosis among patients treated with BTK inhibitors is high. The central nervous system was involved in 25–40% of cases [70]. Furthermore, despite the early introduction of effective antifungal treatment, mortality in this population is high. This highlights the importance of identifying infectious complications, especially fungal ones. Currently, antifungal prophylaxis is not recommended for all patients. However, the introduction of regular screening, particularly using serum markers of fungal infection, or even the implementation of a preventive pharmacologic strategy should be discussed, especially in patients identified as being at highest risk, such as those concomitantly treated with other immunosuppressive drugs or with a history of invasive fungal infection [63]. Finally, there is a CYP3A4 interaction between ibrutinib and voriconazole, the current standard of care for invasive aspergillosis. Studies in patients treated with idelalisib have shown a five-fold increase in the risk of *Pneumocystis jirocevii* pneumonia, justifying the systematic prescription of *Pneumocystis jirocevii* prophylaxis in this population. Impaired responses to vaccination have also been reported in ibrutinib-treated patients [71].

## 5. Phosphatidylinositol 3-Kinase (pi3k) Inhibitors

PI3K inhibitors are orally administered small molecules that inhibit the PI3K signaling pathway, which plays a central role in the development of B lymphocytes and is overexpressed in many lymphoproliferative disorders. Currently, three PI3K inhibitors (idelalisib, duvelisib and umbralisib) are approved for the treatment of chronic lymphocytic leukemia and/or other lymphoid malignancies.

A variety of adverse events have been reported. In particular, inflammatory manifestations such as colitis, hepatitis and pneumonitis often require treatment with high-dose corticosteroids, which increases the risk of infection [14]. In an observational study of 110 patients treated with idelalisib, lower respiratory tract infections were reported in 34.5%, diarrhea in 30.9% and colitis in 9.1% of patients [15].

Neutropenia is also a common AE during the first weeks of idelalisib treatment, occurring in half of patients and in approximately 20% of patients with grade 3–4 neutropenia. Neutropenia is associated with an increased rate of infections, including opportunistic infections (*Pneumocytis jirovecii* pneumonia and CMV reactivations and infections) [72].

*Pneumocystis jirovecii* infections have been reported in up to 3.5% of patients not receiving prophylaxis. Therefore, some guidelines recommended that patients treated with PI3K receive prophylaxis against PJP from the start of treatment until 2–6 months after completion of treatment [33]. Cytomegalovirus (CMV) reactivation occurred in 2.4% of patients during the first six months of treatment [25,72,73,74]. Current expert opinions recommend that CMV serology be performed prior to treatment initiation and that CMV viral load be measured monthly [72]. Acyclovir prophylaxis is also recommended because of the potential for serious skin infections and varicella zoster infections.

## 6. Antiapoptotic Protein BCL-2 Inhibitors

Venetoclax is a potent oral inhibitor of the anti-apoptotic protein BCL-2, which is overexpressed by tumor cells. Venetoclax is currently approved as a single agent or in combination with anti-CD20 monoclonal antibodies for the treatment of chronic lymphocytic leukemia and/or acute myeloid leukemia (AML). The immunosuppressive effect of veneto-clax is associated with cytopenia. Neutropenia occurred in 40–50% of patients treated with venetoclax [20,75]. In a study of 350 patients treated with venetoclax for chronic lymphocytic leukemia, infections of any grade occurred in 72% of patients [20]. The most commonly reported infectious complication was lower respiratory tract infection. In a study of 235 patients receiving venetoclax and hypomethylating agent therapy for acute myeloid leukemia, the overall incidence of bacterial infections was 33.6% and the incidence of probable or confirmed invasive fungal infections was 5.1%.

Venetoclax is metabolized by CY3A4 and therefore has drug–drug interactions with many anti-infectives, including azoles.

## 7. Janus Kinase Inhibitors

Janus kinases (JAKs) are protein tyrosine kinases that bind to transmembrane cytokine receptors and mediate cellular responses to numerous cytokines and growth factors. JAKs phosphorylate sites on the cytoplasmic tail of a variety of hematopoietic and inflammatory cytokine receptors, activating downstream targets via the signal transducer and activator of transcription (STAT) pathway. Through these mechanisms, JAKs play important roles in hematopoiesis and immune cell differentiation.

Ruxolitinib targets JAK1 and JAK2 and induces downregulation of the T helper cell type 1 (Th1) response and cytokines such as interleukin IL-1, IL-6 and tumor necrosis factor α (TNF-α). The most commonly reported adverse events are generally not serious, but an increased risk of serious infectious events has been reported. A systematic review found a high incidence of herpes zoster infections in patients treated with ruxolitinib (OR = 7.39 (95% CI 1.33–41.07)) [76,77]. Whenever possible, patients should be vaccinated against herpes zoster before starting a JAK inhibitor. In addition, patients with complicated herpes zoster or recurrent herpes zoster may be switched to an alternative therapy, or the patient may be treated with daily suppressive antiviral therapy indefinitely if the JAK inhibitor needs to be restarted. In a study of 1144 patients, the most common infectious complications were herpes zoster (8%), bronchitis (6.1%) and urinary tract infections (6%). Rare cases of opportunistic infections (mycobacterial infections, *Pneumocystis jirovecii* pneumonia, invasive fungal infections, PML, disseminated cryptococcosis, HBV reactivation) have also been reported [25,78,79,80]. We may suggest that patients receiving Janus kinase inhibitor therapy be screened for chronic HBV infection or latent tuberculosis prior to initiation of therapy.

## 8. CAR-T Cell Therapy

Adoptive cellular therapy (ACT) has traditionally referred to three different approaches: tumor-infiltrating lymphocyte (TIL) infusion; genetically modified T cell receptor (TCR) therapy; and chimeric antigen receptor (CAR)-modified T cells (CAR-T cells) [81].

CAR-T cells are lymphocytes that have been genetically modified to produce a CAR that specifically targets tumor cell antigens [82,83]. CAR-T cells therapies have produced impressive initial responses in patients with refractory B-cell acute lymphoblastic leukemia [84,85]. CAR T-cell therapy is currently approved for the treatment of diffuse large B-cell lymphoma, acute lymphoblastic leukemia, mantle cell lymphoma, and multiple myeloma [22,23,24,86,87]. Despite excellent anti-malignant activity, adverse events are common with CAR T-cell therapy and include cytokine release syndrome (CRS) (77–93%), neurotoxicity or neurologic events (40–64%), neutropenia (53–87%), and grade 3 or 4 infections (10–31%) [22,23,24].

Most patients undergoing CAR-T cell therapy are at risk of infection (intensive care unit admission, presence of a central venous catheter, prolonged cytopenias). Risk factors for infection have been identified as the presence of severe cytokine release syndrome, the use of multiple lines of treatment prior to CAR-T cell prescription, and the prescription of high doses of CAR-T cells. According to published studies, most infections occur early after CAR-T cell infusion. Bacterial infections are the most common, while fungal infections appear to be rare. The reported viral infections are mainly related to viral reactivations, especially gastrointestinal viruses such as adenovirus, but few respiratory viruses.

### 8.1. Bacterial Infections

Infections following CAR-T cell infusion are common, but their microbiological diagnosis is challenging. In fact, only 72% of infections are microbiologically documented [88]. Most patients undergoing CAR-T cell therapy have often received multiple lines of prior antibiotic therapy and are therefore at risk of colonization and infection with multi-drug resistant bacteria, especially during the neutropenic phase. A recent study showed that 40% of infections occur within the first 90 days after CAR-T cell infusion [89]. In addition, there appears to be a high rate of Clostridioides difficile-related infections in the community, with infection rates ranging from 12.5% to 20% [90,91,92].

Several risk factors are associated with the occurrence of severe bacterial infection. These include severe CRS, neurotoxicity, use of tocilizumab and corticosteroids, and bridging therapy [91]. In addition, failure to respond to CAR-T cell therapy appears to be a strong predictor of severe bacterial infection [88].

### 8.2. Viral Infections

In contrast to bacterial pathogens, viruses are more common later in the course of CAR-T cell therapy. After the first month following CAR-T cell inoculation, lymphopenia (either B- or T-lymphocyte) and hypogammaglobulinemia occur, exposing patients to infectious risks, particularly viral risks. Viral infections typically include respiratory syncytial virus, cytomegalovirus, influenza, and polyomaviruses [91,92,93]. The incidence of viral infections after the first month following CAR-T cell infusion ranged from 9.2 to 28%. In addition, many patients have profound CD4 lymphopenia associated with B-cell aplasia, and reactivation of herpesviruses is frequently observed 6–12 months after CAR-T cell infusion [94]. In addition, cytomegalovirus reactivation has been reported in 1–2% of patients. These data are probably underestimated because most centers do not monitor CMV viral replication in patients undergoing CAR-T cell therapy [67,95]. More recently, it has been shown that patients with hematologic malignancies, especially those treated with CAR-T cell therapy, are at risk for severe forms of SARS-CoV-2 respiratory infection [96,97]. In a study of 57 patients with SARS-CoV-2 infection, 39.3% had a severe form of the infection and the mortality rate was 50%. Lymphopenia was the factor statistically associated with severe infection [98].

In addition, in the population, the viral shedding time of SARS-CoV-2 virus in patients receiving CAR-T cells could be up to two months [99].

### 8.3. Fungal Infections

Despite the high degree of multifactorial immune suppression, fungal infections have been rarely reported in patients receiving CAR-T cell therapy [100], with an incidence ranging from 1 to 5% [100,101,102]. Most fungal infections occur early in the period of initial neutropenia or CRS and are mainly candidemia [102]. Several species of molds have been observed to cause lung disease (*Aspergillus* spp., *Fusarium* spp., *Mucorales*). The most important risk factors for fungal infections are the duration of neutropenia and the prolonged course of systemic corticosteroids prescribed for severe adverse reactions.

### 8.4. Prevention Strategies

Recommendations for prophylaxis and management strategies for infections after CAR T-cell therapy are largely based on guidelines used for HSCT recipients [103,104].

Antibacterial prophylaxis: Most bacterial infections are secondary to the onset of neutropenia and are often related to the depth and duration of neutropenia. The use of granulocyte colony-stimulating factors to shorten the duration of neutropenia in combination with antibiotic prophylaxis has been widely debated.

There have been some concerns that G-CSF may interfere with the CAR T cell response or worsen cytokine release syndrome by activating myeloid-related cytokines [105,106]. Currently, most recommendations are to consider the use of G-CSF only in patients with prolonged neutropenia [84,107]. For example, studies on a possible adverse effect of G-CSF by exacerbation of cytokine release syndrome have shown that its prescription two weeks after CAR-T cell infusion is safe [108].

Antiviral Prophylaxis: Acyclovir prophylaxis is recommended from the start of lymphodepleting chemotherapy and is usually prescribed for at least 3–6 months after CAR-T cell therapy [88]. This duration of prophylaxis is controversial, as cases of herpes virus reactivation have been reported some time after CAR-T cell therapy [92]. For patients with hepatitis B virus (positive HbS antigen or positive anti-HbC antibody alone), it is important to ensure the absence of viral replication prior to CAR-T cell therapy, and antiviral prophylaxis should be administered for at least 6 months and associated with close monitoring of liver enzymes and/or HBV replication.

Given the significant risk of severe SARS-CoV-2 pulmonary infection in the CAR-T cell therapy patient population, many prescribe pre-exposure prophylaxis with monoclonal antibodies (tixagevimab/cilgavimab) despite their lower efficacy against the omicron variant [109].

Antifungal prophylaxis: Because fungal infections are uncommon in patients undergoing CAR-T cell therapy, antifungal prophylaxis is not routine. Thus, for low-risk patients with no history of invasive fungal infections, treatment with fluconazole is most commonly prescribed during the neutropenia period. On the other hand, for high-risk patients with a history of previous fungal infections or high-grade CAR-T cell-associated complications, later-generation antifungal azoles may be indicated [101,104,110]. Trimethoprim/sulfamethoxazole is currently recommended as the gold standard for prophylactic treatment of *Pneumocystis jirovecii* infection and should be initiated approximately one month after CAR-T cell infusion.

Vaccination: Patients undergoing CAR-T cell therapy have significant immune dysregulation, affecting innate immunity in the early phase and both humoral and cellular adaptive immunity in the later phase. A lower rate of seroprotection after vaccination in patients treated with CAR-T cell infusion and its large inter-individual variability argues for the systematic implementation of vaccinations. It is recommended to start vaccination with killed or inactivated vaccines 3 to 6 months after CAR-T cell treatment and to delay the administration of live vaccines until 12 months after CAR-T cell infusion [111].

## 9. Conclusions

New targeted therapies have revolutionized the treatment of hematologic and solid organ malignancies. A high proportion of patients treated with these targeted therapies experience infectious complications, sometimes secondary to the management of side effects. Screening for latent infections and individualized prophylaxis may be advisable.

## Figures and Tables

**Table 1:** Adverse effects and frequencies of new targeted therapies.

Treatment	Adverse Effects					
	Infection (Grade ≥ 3)	Neutropenia	Diarrhea	Hypertension	Hemorrhage/Bleeding	References
Immune checkpoint inhibitor	2–7%	-	1–25%	-	-	[2,3,4]
BTK inhibitors						
Ibrutinib	11–48%	4–17%	5–68%	5–22%	3–15%	[5,6,7,8]
Zanubrutinib	27%	36%	23%	12%	3%	[9]
Acalabrutinib	18%	12%	37%	8%	4%	[10]
Orelabrutinib	15%	29%	7%	-	1%	[11]
Fenebrutinib	17%	4%	29%	-	1%	[12]
PI3K inhibitors						
Idelalisib	20–35%	56%	30–45%	-	-	[13,14,15]
Duvelisib	51–68%	26–50%	43–52%	-	-	[16,17]
Umbralisib	-	14–35%	26–43%	-	-	[18,19]
Anti-apoptotic protein BCL-2 inhibitors	70–75%	40–50%	41%	-	-	[20]
Janus Kinase inhibitors	30–35%	-	-	-	-	[21]
CAR-T cell therapy	10–31%	53–87%	-	-	-	[22,23,24]

BTK: Bruton’s Tyrosine Kinase, PI3K: Phosphatidylinositol 3-kinase, JAK: Janus Kinase.

**Table 2:** Risk infections with new targeted therapies (Adapted from [25]).

Treatment	Drugs	Approved Indications	Infectious Complications	Prophylaxis Suggestions
Immune checkpoint inhibitor				
CTLA-4 targeted agents	Ipilimumab Tremelimumab	Melanoma	Does not appear independently associated with the occurrence of infection but combination with treatment (corticosteroids and/or TNF-α) for irAEs increased infectious risk	Anti-*Pneumocystis* prophylaxis for patients who are expected to receive 20 mg of prednisone daily for at least 4 weeks Hepatitis B and C: prophylaxis or therapy if needed.
(PD)-1 and (PD-L1) targeted agents	Nivolumab Pembrolizumab Atezolizumab	Melanoma Non-small cell lung cancer Head and neck carcinoma Hodgkin lymphoma Metastatic renal cell carcinoma (nivolumab) Urothelial carcinoma and lung cancer (atezolizumab)		
BTK inhibitor	Ibrutinib Acalabrutinib Zanubrutinib	Mantle cell lymphoma Chronic lymphocytic leukemia Waldenström macroglobulinemia Marginal zone lymphoma	Fungal infections: Aspergillus, *Pneumocystis jirovecii*	Assess antifungal prophylaxis or screening for fungal infections Anti-*Pneumocystis* prophylaxis in patients treated with corticosteroids
			Bacterial infections: *Staphylococcus aureus Mycobacterium tuberculosis*	
PI3K inhibitors	Idelalisib Duvelisib Umbralisib	Chronic lymphocytic leukemia Lymphoid malignancies	Fungal infections: *Pneumocystis jirovecii*	CMV serology be performed prior to treatment initiation and that CMV viral load be measured monthly. Acyclovir prophylaxis is also recommended
			Viral infections: CMV, HSV and VZV reactivation	
Antiapoptotic protein BCL-2 inhibitors	Venetoclax	Chronic lymphocytic leukemia Acute myeloid leukemia	Bacterial infections	
			Fungal infections	
JAK inhibitors		Myeloproliferative neoplasms	Bacterial infections: mycobacterial infections	Chronic HBV infection and latent tuberculosis screening
			Fungal infections: *Pneumocystis jirovecii, Cryptococcus* spp.	
			Viral infections: HSV, VZV, JC virus, HBV reactivation	
CAR-T cells		Large B-cell lymphoma Acute lymphoblastic leukemia Mantle cell lymphoma Multiple myeloma	Fungal infections: *Aspergillus* spp., *Fusarium* spp., *Mucorales, Pneumocystis jirovecii*	Anti-*Pneumocystis* prophylaxis (trimethoprim/sulfamethoxazole) Assess antifungal prophylaxis or screening for fungal infections
			Bacterial infections including *Clostridioides difficile* infections	G-CSF in case of prolonged neutropenia
			Viral infections: respiratory syncytial virus, cytomegalovirus, influenza, polyomaviruses, SARS-CoV-2	Acyclovir for at least 3–6 months after CAR-T cell therapy Antiviral therapy for hepatitis B virus in case of positive HbS Ag or AntiHbC Ac alone

BTK: Bruton’s Tyrosin Kinase, CMV: Cytomegalovirus, CTLA-4: Cytotoxic-T-lymphocyte-Antigen 4, G-CSF: Granulocyte Colony Stimulating Factor, HBV: Hepatitis B virus, HSV: Herpes Simplex Virus, irAEs: immune related adverse events, JAK: Janus Kinase, JC: John Cunningham, PD-1: Programmed cell death protein 1, PD-L1: Programmed cell death protein ligand-1 PI3K: Phosphatidylinositol 3-kinase, VZV: Varicella Zona Virus.
